# Abuse of Girls During Childhood and Its Impacts on the Health of Their Adult Lives: A Systematic Review

**DOI:** 10.7759/cureus.34981

**Published:** 2023-02-14

**Authors:** Kyriaki Liveri, Maria Dagla, Antigoni Sarantaki, Eirini Orovou, Evangelia Antoniou

**Affiliations:** 1 Department of Midwifery, University of West Attica, Athens, GRC; 2 Department of Midwifery, University of Western Macedonia, Ptolemaida, GRC

**Keywords:** woman’s physical health, woman’s mental health, child violence, domestic violence, child abuse

## Abstract

Child abuse is a global problem for public health as it negatively affects people and society. The US Centers for Disease Control and Prevention (CDC) associates the adverse experience during childhood with a series of long-term impacts on health. The aim of this study is to explore the impact of child abuse on females’ health, including physical, mental, and social health. The methodology used in this specific review is to carry out a systematic search in electronic databases (Google Scholar, Scopus, PubMed, and Crossref) in published articles between 2004 and 2021. The exclusion criteria were all review papers, such as literature reviews, systematic reviews, and meta-analyses. We also excluded papers that were not written in the English language. Consequently, the inclusion criteria were written in English, original articles, and prospective, case-control, cross-sectional studies that investigated childhood abuse of girls and the health effects in adulthood. Initially, from a total of 796 papers returned by the search, 415 were rejected due to duplicate articles, systematic reviews, and meta-analyses. In addition, 316 articles were rejected due to nonrelevance to the study’s subject. However, from the first 796 papers, 18 met the conditions to be included in the review. We found that females exposed to childhood abuse were more likely to suffer from eating disorders, depression, post-traumatic stress disorder (PTSD), obsessive-compulsive disorder, anxiety, phobias, paranoid ideation and psychoticism, early menarche, sleep disorders, metabolism disorders, cardiovascular diseases, asthma, chronic pain, and early mortality, which are physical and mental conditions in females’ adulthood related to child abuse. The conclusions of this work show that it is a primary need to give emphasis on combating child abuse and timely management when this is a fact.

## Introduction and background

Child abuse is a social phenomenon of concern for public health at a global level [1]. It is defined as any action or omission by the parent or other carer or whatever harms health, the possibility of causing harm, or the threat of harm to a child including physical, sexual, or psychological abuse; neglect; and intimate partner violence [2]. There are more than 4,000,000 reports on an annual basis to child protection agencies concerning more than 4,300,000 children, as a report may include more than one child. The USA has one of the worst records among the industrial countries with five children’s lives on average being lost on a daily basis due to abuse or neglect. The 2019 data mention more than 656,000 victims of child abuse reported to the US state agencies [2,3].

There is a number of studies pertaining to child abuse’s consequences on the later health of the person; however, these studies focus on specific health issues [4-9]. The aim of this study is to investigate the impact of child abuse on the health of adult females, including physical, mental, and social health. To achieve the purpose of this study, a systematic review will be carried out so as to include the studies pertaining to child abuse consequences on the health of females at all levels (physical, mental, and social health).

According to the World Health Organization (WHO) data, approximately three in four children all over the world have suffered physical punishment and/or psychological violence by their parents and carers. Furthermore, one in five females and one in 13 males have stated that they have suffered sexual abuse during childhood up to 17 years old, and 120,000,000 girls and young females under the age of 20 have suffered some form of forced sexual intercourse. However, child abuse is often hidden [9]. Thus, only a small percentage of child victims of violence ultimately receive support from health professionals [10].

The Centers for Disease Control and Prevention (CDC) associates adverse experiences during childhood (including other family dysfunctions apart from abuse and neglect) with a series of long-term impacts on health. The average life expectancy of the ones reporting six or more adverse experiences during childhood was two decades less than the ones reporting none. Ischemic heart disease (IHD), chronic obstructive pulmonary disease (COPD), hepatic disease, and other health-related issues affecting the quality of life are linked to child abuse [2,11-13]. Therefore, preventing these negative experiences could potentially reduce many future health conditions [12].

Apart from the above physical health-related data, it has been documented that traumatic experiences in a child’s life affect the child’s later mental health [14], as well as functionality [15]. People that have suffered physical abuse as children face elevated levels of stress in their adult lives [16] and more general psycho-emotional problems, post-traumatic stress disorder (PTSD), suicide attempts, anxiety disorders, and depression [7].

The aim of this study is to explore the impact of child abuse on females’ health, including physical, mental, and social health, through the systematic search from the international literature.

## Review

Materials and methods

To explore the impacts of child abuse on females’ lives, a systematic search was carried out from the bibliography of international databases (Google Scholar, Scopus, PubMed, and Crossref) according to the Preferred Reporting Items for Systematic Reviews and Meta-Analyses (PRISMA) method [17]. The inclusion criteria of the studies are as follows: a) published in the last 20 years and b) the sample of each study to include impacts of childhood trauma on the children’s adulthood. The exclusion criteria include the following: a) reviews, systematic reviews, and meta-analyses; b) studies not related to adult females (e.g., articles on male trauma); c) incomplete text; and d) articles in other languages apart from English. Additional inclusion criteria were written in English, original articles, and prospective, case-control, cross-sectional studies that investigated childhood abuse of girls and the health effects in adulthood. The search was carried out by three independent researchers (KL, EO, and EA), and no disagreements were seen regarding the results obtained.

The search words were as follows: child abuse OR child violence OR girl abuse AND woman’s health OR woman’s behavior, Child abuse OR child violence OR girl abuse AND the effects on woman’s health, child abuse OR child violence OR girl abuse AND the effects on woman’s mental health, child abuse OR child violence OR girl abuse AND woman’s social health, and child abuse OR child violence AND woman’s physical health.

**Figure 1:**
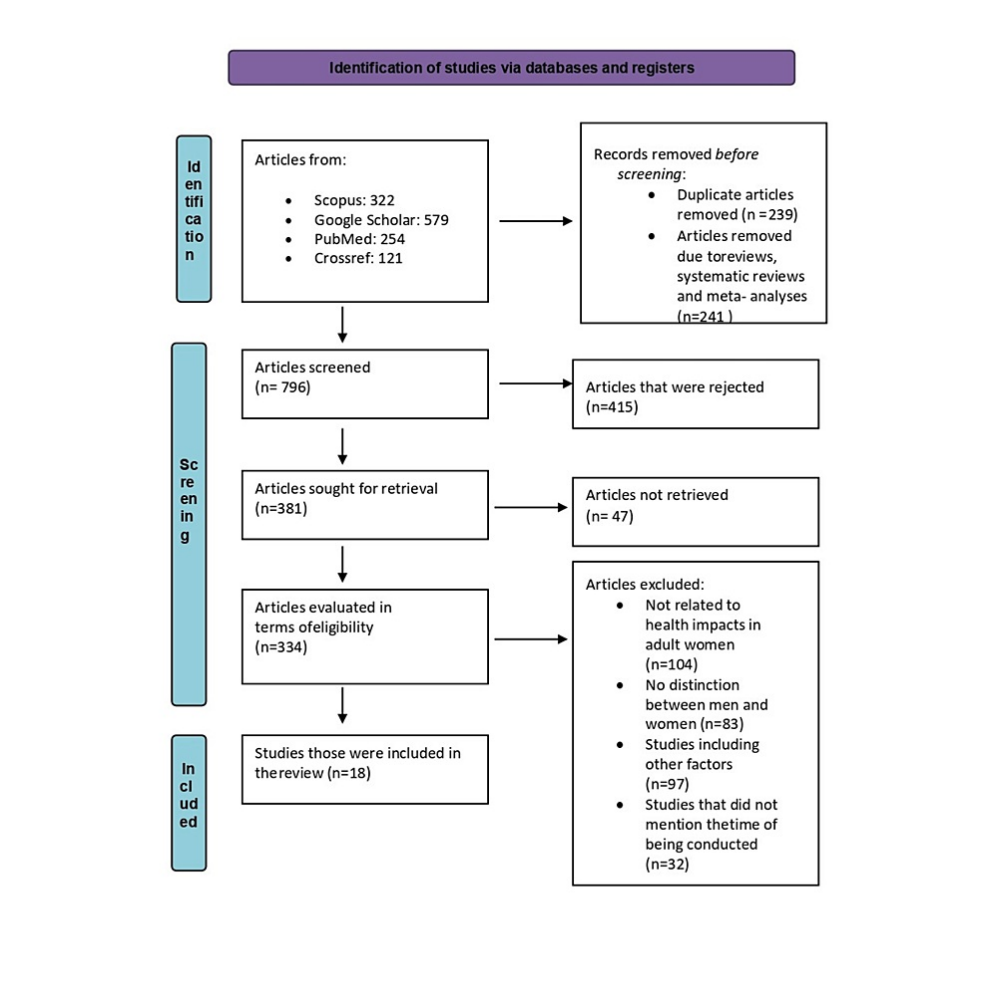
Flow diagram of the research strategy according to PRISMA PRISMA: Preferred Reporting Items for Systematic Reviews and Meta-Analyses

The following flow chart (Figure 1) describes the strategy of selecting studies included in the specific systematic review. The search for studies was carried out in articles published in the 2004-2021 period. Only 18 articles from 1,276 found were included. More specifically, the research articles found via the initial review were shown for the first time only by title and abstract. At first, 480 articles were rejected due to double entries and the fact that they were not written in English but in other languages. Furthermore, 47 articles were rejected as it was impossible to be downloaded. Finally, 334 articles were evaluated in terms of eligibility, 104 of which were rejected because the health impacts did not concern adult females; 83 articles were rejected as there was no distinction between males and females; 97 articles were rejected because they included other factors as well; and finally, 32 were rejected as their date of performance was not mentioned.

As for the methodological quality of the articles (Table 1), nine criteria were classified into three groups (selection, comparability, and results). In selection, the first criterion is concerned with the representative sample of those exposed. The second criterion is concerned with the choice of not exposing the sample. The third criterion was whether there were findings of exposure, and the fourth criterion is concerned with the outcome not preceding the study. In comparability, the first criterion is concerned with adjustment for educational level, and the second criterion involved adjustment for an additional confounder. In results, the first criterion is concerned with the evaluation of the results of each study. The second criterion is related to sufficient follow-up time of the sample, and the third criterion was about the non-bias of the articles.

**Table 1:** Methodological quality of the articles All nine criteria were a) representative exposure sample, b) selection of nonexposed, c) exposure finding, d) outcome not preceding the study, e) adaptation for educational level, f) adaptation for additional confounding factor, g) outcome evaluation, h) adequate monitoring time, and i) non-bias of wear. The symbol “*” means that the study met the specific criterion, and the symbol “-” means that the study did not meet it. Selection has four criteria (a, b, c, and d). Comparability has two criteria (e and f). Results has three criteria (g, h, and i)

Authors (year)	Selection	Comparability	Result	Total
Afifi et al. (2017) [4]	* - * *	* *	* - *	7
Alvarez et al. (2007) [5]	* - * *	* -	* * *	7
Banerjee et al. (2018) [6]	- - * *	* -	* - *	5
Boynton-Jarrett et al. (2021) [18]	* - * *	* *	* * *	8
Cannon et al. (2010) [19]	- - * *	* -	* - *	5
Chen et al. (2016) [20]	- - * *	* -	* * *	6
Lang et al. (2008) [21]	- - * *	* *	* - *	6
Cozier et al. (2021) [8]	* - * *	* -	* - *	6
Lee et al. (2017) [22]	- * * *	* -	* - *	7
Lundqvist et al. (2004) [23]	- - * *	* *	* * *	7
Mason et al. (2013) [24]	* * * *	* *	* * *	9
Midei et al. (2013) [9]	- - * *	* -	* - *	5
Riley et al. (2010) [25]	* - * *	* -	* * *	7
Thurston et al. (2014) [26]	- - * *	* -	* - *	5
Thurston et al. (2017) [27]	- - * *	* -	* * *	6
Walsh et al. (2007) [28]	- - * *	* -	* - *	5
Wise et al. (2009) [29]	* - * *	* -	* * *	7
Zhong et al. (2016) [30]	- - * *	* *	* - *	6

Results

Of the eighteen studies included in the systematic review, 17 were carried out in the American continent (14 in the USA, two in Peru, and one in Canada) and only one in Sweden. In terms of the design of the studies included, seven of them were cross-sectional studies, seven were cohort studies (one was a prospective cohort study), and the remaining four were case-control studies (Table 2).

**Table 2:** Studies included in the review MIDUS: Midlife in the United States

Authors (year and country)	Design	Population	N	Data	Exposure	Results
Afifi et al. (2017, USA) [4]	Cross-sectional study	Adult US residents	36,309	National Epidemiologic Survey on Alcohol and Related Conditions-III (NESARC-III): questionnaire and interview	Child abuse (harsh physical punishment and physical, sexual, or emotional abuse)	Eating disorders (anorexia nervosa, bulimia nervosa, and binge‐eating disorder)
Alvarez et al. (2007, USA) [5]	Cross-sectional study	Non-pregnant females over the age of 18	11,115	California Women’s Health Survey: telephone interview	Child abuse (physical and sexual abuse)	Obesity
Banerjee et al. (2018, Peru) [6]	Cross-sectional study	Pregnant Peruvian females participating in the cohort and Pregnancy Outcomes, Maternal and Infant Cohort Study (PrOMIS)	3,372	Pregnancy Outcomes, Maternal and Infant Cohort Study (PrOMIS): interview based on structured questionnaire	Child physical and sexual abuse	Asthma
Boynton-Jarrett et al. (2021, USA) [18]	Prospective cohort study	Females having participated in the Black Women’s Health Study in 1995, aged 21-69	29,998	Black Women’s Health Study: self-managed questionnaire	Child physical and sexual abuse	Sleep disorder (sleep apnea)
Cannon et al. (2010, USA) [19]	Case-control study	English-speaking females aged 18-64	3,568	Group Health Cooperative: telephone interview	Child abuse exposure as a witness during childhood	High prevalence of depression, domestic violence, and higher use of health care and mental health services
Chen et al. (2016, USA) [20]	Case-control study	Adults aged 25-74	6,285	Research on the development of middle age in the USA: questionnaire and National Death Index	Physical and emotional abuse in childhood	Mortality
Lang et al. (2008, USA) [21]	Case-control study	Females in primary care clinic of the Veterans Affairs San Diego Health System (VASDHS) in 1998	200	Veterans Affairs San Diego Health System (VASDHS): self-report measures	Child emotional, physical, and sexual abuse	Post-traumatic stress disorder (PTSD)
Cozier et al. (2021, USA) [8]	Cohort study	Black females, aged 21-69	36,152	Black Women’s Health Study: two-year observation questionnaires	Physical and sexual abuse during childhood	Systemic lupus erythematosus (SLE)
Lee et al. (2017, USA) [22]	Cohort study	English-speaking females in the USA, aged 25-78	1,225	Biomarker MIDUS: Telephone observation research and self-assessment questionnaires	Emotional abuse, physical abuse, and sexual abuse in childhood	Biological dysfunction
Lundqvist et al. (2004, Sweden) [23]	Cohort study	Females having suffered sexual abuse during childhood (0-18 years); they remember it and know who the culprit was without psychosis diagnosis or ongoing substance abuse	45	Department of Psychiatry, Lund University Hospital: interview and questionnaire	Child sexual abuse	Psychiatric symptoms (depression, obsessive-compulsive disorder, anxiety, phobias, paranoid ideation, and psychoticism)
Mason et al. (2013, USA) [24]	Cross-sectional study	Registered female nurses hired at the age of 25-42 years	57,321	Nurses’ Health Study II: questionnaire	Physical and sexual abuse during childhood	Food addiction
Midei et al. (2013, USA) [9]	Cohort study	Premenstrual or early perimenstrual females in middle age	342	Pittsburgh area from the Study of Women’s Health Across the Nation (SWAN): questionnaire and blood tests	Child physical, sexual, and emotional abuse	Metabolic syndrome
Riley et al. (2010, USA) [25]	Cohort study	Females participating in the Nurses’ Health Study II, aged 25-44	68,505	Questionnaires and hypertension recording by a physician every two years	Child physical and sexual abuse	Hypertension
Thurston et al. (2014, USA) [26]	Cohort study	Females aged 42-52, with uterus and ≥1 ovary, non-pregnant or breast-feeding, nonuse of oral contraceptive or hormone therapy, and ≥1 menstrual cycle in the previous three months	1,402	Study of Women’s Health across the Nation (SWAN): physical and sexual abuse score via questionnaire, blood screening, physical measurements, and ultrasound of carotid artery	Child physical and sexual abuse	Higher intima-media thickness (IMT)
Thurston et al. (2017, USA) [27]	Cross-sectional study	Perimenstrual and post-menstrual females (19), nonsmokers, aged 40-60	295	MsHeart study: questionnaire on childhood trauma, sleep monitoring, blood screen, and carotid ultrasound (intima-media thickness {IMT} and plaque)	Child emotional, physical, and sexual abuse	Intima-media thickness (IMT) and carotid plaque
Walsh et al. (2007, Canada) [28]	Cross-sectional study	Females over the age of 15 staying in private houses	3,381	Ontario Health Survey	Physical abuse and sexual abuse during childhood	Chronic pain
Wise et al. (2009, USA) [29]	Case-control study	Self-defined black females that had submitted participation in the Black Women’s Health Study in the USA	35,330	Black Women’s Health Study: questionnaire	Physical abuse and sexual abuse during childhood	Early menarche
Zhong et al. (2016, Peru) [30]	Cross-sectional study	Pregnant females	2,964	Prenatal clinics	Childhood abuse	Suicidal ideation

The types of abuse involved a) physical abuse, which includes beating, burning, kicking, punching, biting, maiming or killing, or the use of objects or weapons; b) sexual abuse, which includes sex of any kind, sex trafficking, exhibitionism or exposing oneself to a minor, obscene conversations, phone calls, text messages, or digital interaction; and c) emotional abuse, which includes constant criticism, threats, or rejection, as well as withholding love, support, or guidance.

According to our results, there is a strong correlation between child abuse and eating disorders in adulthood. More specifically, we identified that anorexia nervosa, bulimia nervosa, binge‐eating disorder, and obesity are associated with all types of child abuse, with rates ranging between 2.7% and 8% in females [4,5].

In addition, among the included articles, serious psychosocial data were found. Exposure to child abuse, even as a witness to the events, can result in the victimization of the female and exposure to domestic violence, depression [18,19] and PTSD [20,21] and other psychiatric symptoms, such as obsessive-compulsive disorder, anxiety, phobias, paranoid ideation, and psychoticism [20-24].

Furthermore, mental disorders can affect physical health. Females who had experienced childhood violence were more likely to make increased use of health services and have poor health status [19]. In more details, we found that higher severity of physical and/or sexual abuse was associated with sleep disorders (sleep apnea) [18], systemic lupus erythematosus (SLE) [8], imbalanced secretion of stress hormones, lipid and glucose metabolism disorders [9,22], cardiovascular diseases [22,25], intima-media thickness and carotid plaque [26-28], asthma [6], and chronic pain [28,29], which is consequently observed after physical violence. Nevertheless, females reporting emotional or physical abuse run higher risk of premature mortality. This causes concern for cardiovascular diseases, cancers, depression, and the use of substances [20].

Discussion

The results of our study show that eating disorders, depression, PTSD, obsessive-compulsive disorder, anxiety, phobias, paranoid ideation and psychoticism, early menarche, food addition, sleep disorders, metabolism disorders, cardiovascular diseases, asthma, chronic pain, suicidal ideation, and early mortality were related to child abuse. Also, the increased use of health services and, more specifically, mental health services is related not only to increased morbidity but also to anxiety and phobic disorders.

More specifically, according to the results of our study, the various types of child abuse (harsh physical punishment, physical abuse, sexual abuse, emotional abuse, emotional neglect, physical neglect, and exposure to violence) are related to eating disorders [4] and cardiovascular disorders [25]. In particular, physical and/or sexual abuse is related to obesity [5], asthma [6], chronic pain [28], increased possibility of SLE occurrence [8], metabolic syndrome [9], and higher intima-media thickness, meaning a higher likelihood of cardiovascular conditions, even more for females sleeping less than six hours/night or having hot flushes [27], as well as early menarche [29]. Sexual abuse affects mainly females’ mental health. Suicidal ideation during the perinatal period has been directly related to childhood abuse [30]. Also, psychiatric symptoms such as depression, PTSD, obsessive-compulsive disorder, anxiety, phobias, paranoid ideation, and psychoticism were related to sexual trauma. Several studies suggest the impact of childhood abuse on a person’s long-term physical health. For example, back pain, nightmares, headaches, chronic pain, eating disorders, frequent fatigue, sleep problems, chest pain, feeling of suffocation, urination problems, diarrhea, constipation, severe bruising, and shortness of breath were associated with a history of child abuse [31,32].

As for the long-term effects of that childhood abuse on mental health, the literature shows that is positively related to adult aggression, depression, hostility, anger, fear, anxiety disorders, and personality disorders [33,34]. The link between childhood abuse and psychological effects in adults is well-documented; however, four potential pathways (emotional, behavioral, social, and cognitive) have been described that articulate the impact of childhood maltreatment on adult health [35].

Finally, in terms of the impacts of child abuse on the social health of adult females, the research showed that there is an increased likelihood to be victims of domestic violence and social inclusion problems. The association between childhood abuse and intimate partner violence in adulthood is a well-known phenomenon [36], which is mainly based on the individual’s memory images and the resulting low self-esteem. Low self-esteem, as it turns out, results in the development of depression, anxiety, and stress [37]. Although this study is the first to systematically examine the effects of childhood abuse on females’ health, there are some important limitations. No study examined major confounders, and also, we do not know in all studies the size of the child’s trauma, the child’s response, and who played the role of abuser. In addition, another limitation of this study concerns the almost exclusive conduct of the studies in the USA. However, it would be quite interesting to see the effects of childhood abuse on females’ adulthood in global populations.

## Conclusions

This research looked into child abuse from the side of impacts on the physical and mental health of females and their relationships. The results showed a plethora of severe problems brought by child abuse upon all aspects of the health of adult females. Although the objective is the “physical and mental health of females having suffered abuse in their childhood,” it is expected to assume that females’ social health is also affected. Females that reported emotional and medium physical abuse have an increased mortality risk from all causes in the observation period, something which is confirmed by a few studies that have focused on it. The conclusions of this work show that it is a primary need to give emphasis on combating child abuse and timely management when this is a fact. Furthermore, it is important for child abuse victims to receive special biological-mental-social health to manage early any impacts of the various forms of child abuse. Finally, the systemic review of the literature showed that it is extremely important to perform studies on whether the type of child abuse is related to impacts in specific health sectors and also find a mechanism that links the various impacts on the health of adult females having suffered abuse in their childhood.

## Appendices

Table 3 shows the PRISMA 2020 checklist.

**Table 3:** PRISMA 2020 checklist PRISMA: Preferred Reporting Items for Systematic Reviews and Meta-Analyses

Section and topic	Item number	Checklist item	Location where item is reported
Title			
Title	1	Identify the report as a systematic review	Yes
Abstract			
Abstract	2	See the PRISMA 2020 for abstract checklist	Yes
Introduction			
Rationale	3	Describe the rationale for the review in the context of existing knowledge	Yes
Objectives	4	Provide an explicit statement of the objective(s) or question(s) the review addresses	Yes
Methods			
Eligibility criteria	5	Specify the inclusion and exclusion criteria for the review and how studies were grouped for the syntheses	Yes
Information sources	6	Specify all databases, registers, websites, organizations, reference lists, and other sources searched or consulted to identify studies. Specify the date when each source was last searched or consulted	Yes
Search strategy	7	Present the full search strategies for all databases, registers, and websites, including any filters and limits used	Yes
Selection process	8	Specify the methods used to decide whether a study met the inclusion criteria of the review, including how many reviewers screened each record and each report retrieved; whether they worked independently; and, if applicable, details of automation tools used in the process	Yes
Data collection process	9	Specify the methods used to collect data from reports, including how many reviewers collected data from each report; whether they worked independently; any processes for obtaining or confirming data from study investigators; and, if applicable, details of automation tools used in the process	Yes, we added it to the text
Data items	10a	List and define all outcomes for which data were sought. Specify whether all results that were compatible with each outcome domain in each study were sought (e.g., for all measures, time points, and analyses) and, if not, the methods used to decide which results to collect	No
	10b	List and define all other variables for which data were sought (e.g., participant and intervention characteristics and funding sources). Describe any assumptions made about any missing or unclear information	Yes
Study risk of bias assessment	11	Specify the methods used to assess risk of bias in the included studies, including details of the tool(s) used; how many reviewers assessed each study and whether they worked independently; and, if applicable, details of automation tools used in the process	Yes
Effect measures	12	Specify for each outcome the effect measure(s) (e.g., risk ratio and mean difference) used in the synthesis or presentation of results	Yes
Synthesis methods	13a	Describe the processes used to decide which studies were eligible for each synthesis (e.g., tabulating the study intervention characteristics and comparing against the planned groups for each synthesis {item number 5})	Yes
	13b	Describe any methods required to prepare the data for presentation or synthesis, such as handling of missing summary statistics or data conversions	Not applicable
	13c	Describe any methods used to tabulate or visually display results of individual studies and syntheses	Not applicable
	13d	Describe any methods used to synthesize results and provide a rationale for the choice(s). If meta-analysis was performed, describe the model(s) and method(s) to identify the presence and extent of statistical heterogeneity and software package(s) used	Yes
	13e	Describe any methods used to explore possible causes of heterogeneity among study results (e.g. subgroup analysis and meta-regression)	No
	13f	Describe any sensitivity analyses conducted to assess the robustness of the synthesized results	Not applicable
Reporting bias assessment	14	Describe any methods used to assess risk of bias due to missing results in a synthesis (arising from reporting biases)	Yes (Table 1)
Certainty assessment	15	Describe any methods used to assess certainty (or confidence) in the body of evidence for an outcome	Not applicable
Results			
Study selection	16a	Describe the results of the search and selection process, from the number of records identified in the search to the number of studies included in the review, ideally using a flow diagram	Yes
	16b	Cite studies that might appear to meet the inclusion criteria but which were excluded, and explain why they were excluded	It was not mentioned why they were excluded. We simply mentioned them in the manuscript
Study characteristics	17	Cite each included study, and present its characteristics	Yes
Risk of bias in studies	18	Present assessments of risk of bias for each included study	Yes
Results of individual studies	19	For all outcomes, present, for each study, a) summary statistics for each group (where appropriate) and b) an effect estimate and its precision (e.g., confidence/credible interval), ideally using structured tables or plots	Not applicable
Results of syntheses	20a	For each synthesis, briefly summarize the characteristics and risk of bias among contributing studies	Yes
	20b	Present results of all statistical syntheses conducted. If meta-analysis was done, present for each the summary estimate and its precision (e.g., confidence/credible interval) and measures of statistical heterogeneity. If comparing groups, describe the direction of the effect	Not applicable
	20c	Present results of all investigations of possible causes of heterogeneity among study results	Not applicable
	20d	Present results of all sensitivity analyses conducted to assess the robustness of the synthesized results	Not applicable
Reporting biases	21	Present assessments of risk of bias due to missing results (arising from reporting biases) for each synthesis assessed	No
Certainty of evidence	22	Present assessments of certainty (or confidence) in the body of evidence for each outcome assessed	Not applicable
Discussion			
Discussion	23a	Provide a general interpretation of the results in the context of other evidence	Yes
	23b	Discuss any limitations of the evidence included in the review	Yes
	23c	Discuss any limitations of the review processes used	No
	23d	Discuss implications of the results for practice, policy, and future research	Yes
Other information			
Registration and protocol	24a	Provide registration information for the review, including the registered name and registration number, or state that the review was not registered	Not applicable
	24b	Indicate where the review protocol can be accessed, or state that a protocol was not prepared	Not applicable
	24c	Describe and explain any amendments to information provided at registration or in the protocol	Not applicable
Support	25	Describe sources of financial or nonfinancial support for the review and the role of the funders or sponsors in the review	Yes
Competing interests	26	Declare any competing interests of review authors	Yes
Availability of data, code, and other materials	27	Report which of the following are publicly available and where they can be found: template data collection forms, data extracted from included studies, data used for all analyses, analytic code, and any other materials used in the review	Not applicable
